# Antimicrobial activity of Desplac® oral gel in the subgingival multispecies biofilm formation

**DOI:** 10.3389/fmicb.2023.1122051

**Published:** 2023-05-16

**Authors:** Bruno Bueno-Silva, Karyne Rossit Kiausinus, Francisco Jeferson dos Santos Gonçalves, Marcus Vinícius Cintra Moreira, Eder Gonzaga de Oliveira, Aldo Brugnera Junior, Magda Feres, Luciene Cristina Figueiredo

**Affiliations:** ^1^Dental Research Division, Guarulhos University, Guarulhos, Brazil; ^2^Sysplac Produtos Médicos e Odontológicos LTDA, São Paulo, Brazil; ^3^Education College of the European Master in Oral Laser Application (EMDOLA), University of Liège, Liège, Belgium; ^4^Research Collaborator at the IFSC-University of São Paulo (USP), São Paulo, Brazil

**Keywords:** multispecies biofilm, antimicrobial, periodontal disease, natural agents, *Porphyromonas gingivalis*

## Abstract

Natural products are well-known due to their antimicrobial properties. This study aimed to evaluate the antimicrobial effect of Desplac® product (composed of Aloe Vera, Propolis Extract, Green Tea, Cranberry, and Calendula) on the subgingival biofilm. Two different protocols were used to treat the 33-species biofilms: (A) 2×/day (12/12  h) for 1  min with Desplac® or Noplak Toothpaste (Chlorhexidine + Cetylpyridinium Chloride) or Oral B ProGengiva (stannous Fluoride) or a placebo gel; (B) a 12-h use of the Desplac® product or 0.12% chlorhexidine gel or a placebo gel. After 7 days of biofilm formation, the metabolic activity (MA) and biofilm profile were determined by 2,3,5-triphenyltetrazolium chloride and Checker-board DNA–DNA hybridization, respectively. Statistical analysis used the Kruskal-Wallis test followed by Dunn’s post-hoc. In protocol A, all treatments presented reduced MA compared to the placebo (*p* ≤ 0.05). The Desplac®-treated biofilm showed a similar microbial profile to other antimicrobials, although with higher bacterial total counts. In protocol B, MA of Desplac®-treated biofilms was lower than the placebo’s MA but higher than chlorhexidine-treated biofilms (*p* ≤ 0.05). Pathogen levels in Desplac®-treated biofilms were lower than in placebo-treated biofilms and elevated compared to the chlorhexidine-treated biofilms (*p* ≤ 0.05). Desplac® inhibited the biofilm development and disrupted the mature subgingival biofilm, highlighting its effect on *Tannerella forsythia* counts.

## 1. Introduction

The mechanical removal of biofilm is necessary for the prevention, treatment, and post-therapy maintenance of periodontal diseases, either professionally or through manual control by the individual (Axelsson et al., 2004). However, satisfactory cleanliness levels are not always achieved with manual brushing alone. Furthermore, tooth surfaces only represent a small percentage of the total mouth area (Kerr et al., 1991). Therefore, the use of antimicrobial agents can help control the supragingival biofilm because they are able to reach other oral niches and can delay the accumulation on the tooth surface (Teles and Teles, 2009).

One of the ways to use chemical agents in oral health is through mouthwashes. These agents can act by promoting cell death, inhibiting bacterial reproduction, or inhibiting cell metabolism (Tartaglia et al., 2017). A wide range of antimicrobial chemical agents are being studied as active principles to control dental biofilm formation, such as bisbiguanides (chlorhexidine), quaternary ammonium compounds (cetylpyridinium chloride), essential oils, enzymes (mutanase/glucanase, amyloglucosidase/glucose oxidase), metal ions (zinc, copper, tin), and plant extracts (Jones, 1997; Radford et al., 1997; Faveri et al., 2006; Feres and Figueiredo, 2009; Kumar et al., 2013; James et al., 2017).

In this context, previous research (Newman and Cragg, 2020) reported that among all new drugs approved by the US’s Food and Drug Administration (FDA), or other equivalent entities in other countries, 30% are directly derived from natural products, 44% are from derivatives of these natural products, and only 26% have synthetic origins. Natural products have been a more sustainable and ecological therapeutic alternative for different clinical situations. Some of the main benefits arising from the use of natural products are formulas that are not aggressive to the human body, they do not present polluting agents in nature, and they decrease the risk of allergies and inflammatory diseases. The search for natural cosmetic products for dental applications (dentifrices and mouthwashes) has been growing constantly. There is intense research in the literature to find new antimicrobials that lead to the rupture of the subgingival multispecies biofilm and one of the main sources to discover novel compounds are the natural products (Freires et al., 2015; Lazar et al., 2016; Slobodnikova et al., 2016; Arbia et al., 2017; Lee et al., 2017; Miranda et al., 2019; Bim-Júnior et al., 2020; de Figueiredo et al., 2020; de Faveri et al., 2022).

The initial studies to prove the antimicrobial effect of a novel agent usually adopt the biofilms model. The common monospecies biofilms were inappropriate for the periodontal disease since bacteria organize themselves as dynamic multispecies biofilms in the subgingival environment (Prado et al., 2022). Hence, the literature looks for innovative biofilm models to reproduce what happens *in vivo*. Recently, our research group developed a multispecies biofilm composed of 33 distinct bacterial species using the Calgary Biofilm Device, which includes a cover with 96 polystyrene pegs mounted up into a 96-well plate (Miranda et al., 2019; de Figueiredo et al., 2020). This model’s advantages include the number of health- and disease-associated species, encompassing most of the species studied in Socransky’s complexes (Socransky et al., 1998). To our knowledge, no biofilm model quantifies so many species as the present one. It better simulates what happens *in vivo* when compared to a model with fewer species due to the number of representative bacteria species involved in the periodontal disease initiation and progression included in the model. In addition, bacteria must actively adhere to pegs instead of being deposited at the bottom of the wells in order to form the biofilm. Recently, the natural product Desplac® (Premium Oral Gel), composed of propolis, Aloe vera, green tea, cranberry, and calendula, became available in the Brazilian market. This oral product has lawful approval from the responsible government departments in the country (Brazilian Health Regulatory Agency—ANVISA) and recommendations for dental use. The main biological constituents of natural products can act as antioxidants, anti-inflammatories, and antimicrobials, in addition to other properties. Several studies have already been carried out to better understand the use of propolis (Bueno-Silva et al., 2013, 2015, 2017a,b,c, 2020; Lima Cavendish et al., 2015; Kiani et al., 2022), Aloe vera (Sujatha et al., 2014; Ali and Wahbi, 2017; Pattnaik et al., 2022), green tea (Yang et al., 2016; Miyoshi et al., 2020; Kong et al., 2022), cranberry (Ben Lagha et al., 2020; Galarraga-Vinueza et al., 2020; Mizutani et al., 2021; Nawrot-Hadzik et al., 2021a,b; Nemzer et al., 2022), and calendula (Alexandre et al., 2018; Tanideh et al., 2020; Yin et al., 2021).

However, it is necessary to carry out scientific investigations that prove and support the commercial recommendations of these products. Thus, the objective of this study was to evaluate the antimicrobial effect of the Desplac® product (Premium Oral Gel) on the metabolic activity and the profile of multispecies subgingival *in vitro* biofilm model.

## 2. Materials and methods

### 2.1. Experimental design

**Figure 1:**
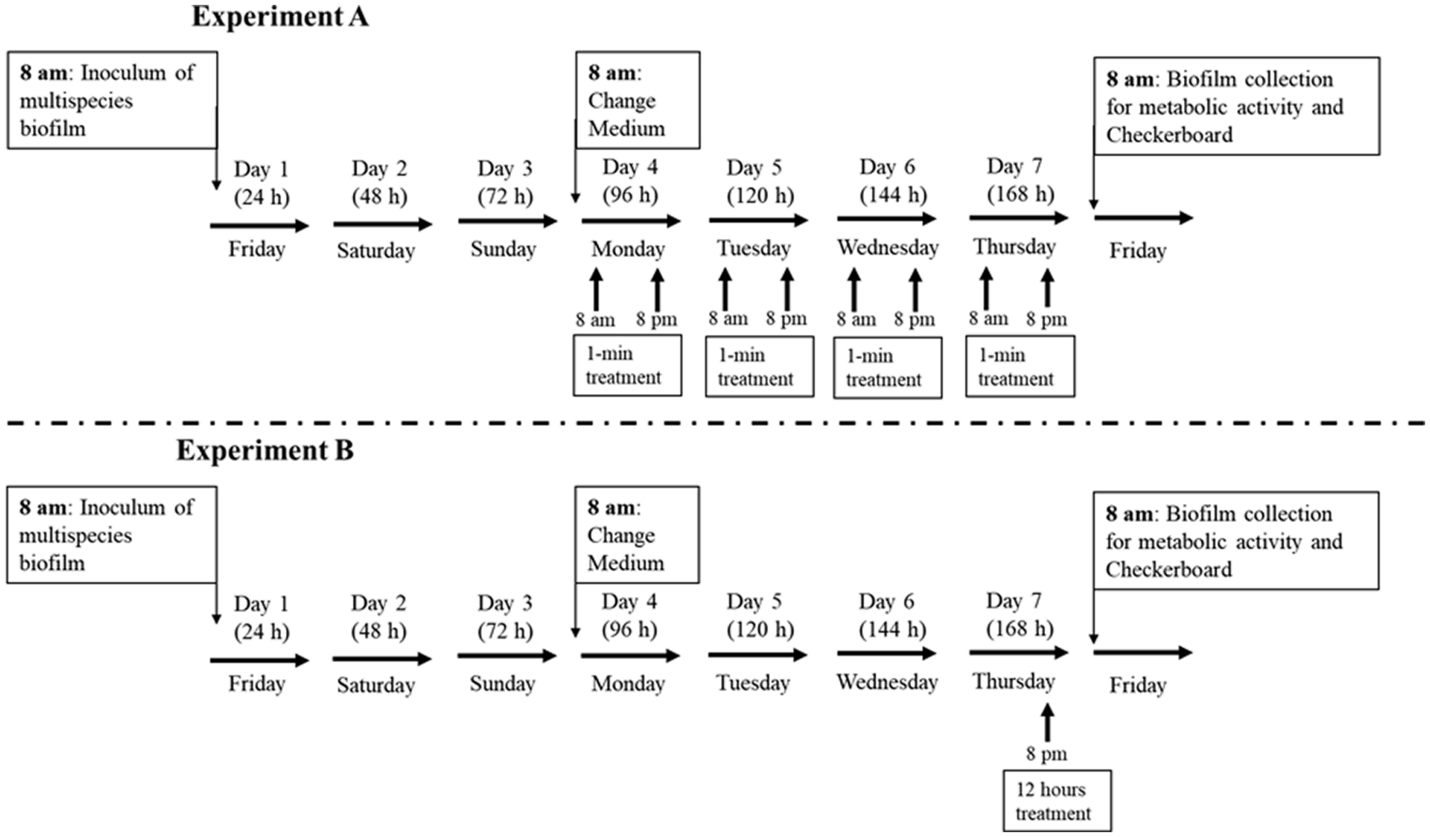
Scheme of the therapeutic approaches of experiments A and B.

The design of this study (Figure 1) involved two laboratory experiments that aimed to reproduce the clinical indications of the Desplac® product (Premium Oral Gel): as a dentifrice (A) and as a night gel on acrylic plates (B). The (*in vitro*) multispecies bacterial biofilm was exposed to the respective products according to the test or control groups.

Experiment A (simulation of use as a toothpaste, 2×/day, 12/12 h, for 1 min).

- Test Group: Desplac® (Premium Oral Gel);
- Negative Control Group: Placebo Gel;
- Positive Control Group 1: Noplak Toothpaste (Chlorhexidine + Cetylpyridine Chloride);
- Positive Control Group 2: Oral B ProGengiva (Stannous Fluoride).

For experiment A, the pins with attached biofilm were removed from the culture media, placed in another 96-well plate with the treatments each time, and later returned to the same media.

Experiment B (simulation of use as an overnight gel on acrylic plates, for 12 h on day 6).

- Test Group: Desplac® (Oral Gel Premium);
- Negative Control Group: Placebo Gel;
- Positive Control Group: 0.12% Chlorhexidine Gel.

The cover with the pins was placed in another 96-well plate containing culture media BHI mixed with the treatments for experiment B. Noplak Toothpaste and Oral B ProGengiva products are commercially available and were purchased locally. The Desplac® and the placebo gels were provided by the company responsible for the former’s manufacturing (Sysplac). The placebo gel was formulated with the same physical characteristics as the Desplac® product but without the active ingredients. Considering that there is no other commercially available dental product in the national market with the same recommendation for overnight use during 12 h, a 0.12% Chlorhexidine Gel, purchased from a compounding pharmacy, was chosen for its recognized gold standard antimicrobial activity.

### 2.2. *In vitro* multispecies biofilm model

*In vitro* multispecies biofilm cultures were prepared with 33 bacterial species (Table 1) as described by Miranda et al. (2020), with some modifications. Tryptone soy agar with 5% sheep blood (Probac, São Paulo, Brazil) was used to grow most species under anaerobic conditions, 85% nitrogen, 10% carbon dioxide, and 5% hydrogen. *Porphyromonas gingivalis* was grown on tryptone soy agar containing yeast extract enriched with 1% hemin, 5% menadione, and 5% sheep blood. *Tannerella forsythia* was grown on tryptone soy agar containing yeast extract enriched with 1% hemin, 5% menadione, 5% sheep blood, and 1% N-acetylmuramic acid. All species were allowed to grow on agar plates for 24 h and then transferred to glass tubes containing Brain Heart Infusion (BHI) culture medium (Becton Dickinson, Sparks, MD, United States) supplemented with 1% hemin. After 24 h growing on conical tubes, the optical density was adjusted for the inoculum to have about 10^8^ cells/mL of each species. A dilution of individual cell suspensions was performed and 100 μL aliquots containing 10^6^ cells from each species were added to 11,700 μL of BHI broth complemented with 1% hemin and 5% sheep blood to obtain an inoculum of 15 mL (Miranda et al., 2019, 2020; Pingueiro et al., 2019; Shibli et al., 2021).

**Table 1:** List of bacterial species cultured in multispecies biofilms.

Species	ATCC
***Actinomyces* sp.**	
*Actinomyces naeslundii*	12104
*Actinomyces oris*	43146
*Actinomyces gerencseriae*	23840
*Actinomyces israelii*	12102
**Purple complex**	
*Veillonella parvula*	10790
*Actinomyces odontolyticus*	17929
**Yellow complex**	
*Streptococcus sanguinis*	10556
*Streptococcus oralis*	35037
*Streptococcus intermedius*	27335
*Streptococcus gordonii*	10558
*Streptococcus mitis*	49456
**Green complex**	
*Aggregatibacter actinomycetemcomitans*	29523
*Capnocytophaga ochracea*	33596
*Capnocytophaga gingivalis*	33624
*Eikenella corrodens*	23834
*Capnocytophaga sputigena*	33612
**Orange complex**	
*Campylobacter showae*	51146
*Campylobacter gracilis*	33236
*Eubacterium nodatum*	33099
*Fusobacterium nucleatum vincentii*	49256
*Parvimonas micra*	33270
*Fusobacterium nucleatum polymorphum*	10953
*Fusobacterium periodonticum*	33693
*Prevotella intermedia*	25611
*Streptococcus constellatus*	27823
**Red complex**	
*Porphyromonas gingivalis*	33277
*Tannerella forsythia*	43037
**Others species**	
*Eubacterium saburreum*	33271
*Streptococcus anginosus*	33397
*Streptococcus mutans*	25175
*Selenomonas noxia*	43541
*Propionibacterium acnes*	11827
*Gemella morbillorum*	27824

The strains were categorized in the microbial complexes described by Socransky et al. (1998).

The multispecies biofilm model was developed using a Calgary biofilm device (CBD) in a 96-well plate (Nunc; Thermo Scientific, Roskilde, Denmark; Ceri et al., 1999). A 150 μL aliquot of each inoculum was added to the wells and corresponded to ~1 × 10^4^cells of each bacterial strain—except for *P. gingivalis* and *Prevotella intermedia*, whose inocula were adjusted to 2 × 10^4^cells. A lid containing polystyrene pins was used to seal the 96-well plate (Nunc TSP system; Thermo Scientific, Roskilde, Denmark). Coated plates were incubated at 37°C under anaerobic conditions. After 72 hours, the used medium (BHI broth with 1% hemin and 5% sheep blood) was replaced and biofilm cultures were kept at 37°C under anaerobic conditions for an additional 4 days to obtain 7-day-old biofilms (Miranda et al., 2019). In the middle of the seventh day, the biofilms were transferred to a culture medium mixed with the different treatments according to the description of Experiments A and B. All products used in the experiments (Desplac®—Premium Oral Gel; Placebo Gel; Noplak Dentifrice; Oral B ProGengiva; Chlorhexidine Gel 0.12%) were diluted (1 part of the product for 2 parts of BHI) to obtain a more fluid solution that could act on the biofilm for its biological properties and not for a merely mechanical effect. After 7 days of biofilm formation, the pins were collected for microbiological processing. The experiments were performed in triplicate for each of the groups (Miranda et al., 2019; Faveri et al., 2022).

#### 2.2.1. Quantification of biofilm bacterial metabolic activity

The effects of Desplac® and other products used as positive and negative controls on the metabolic activity of multispecies biofilm cells were measured in a spectrophotometric assay with 2,3,5-triphenyltetrazolium chloride (TTC; catalog N^o^. 17779; Fluka analytical). TTC is used to differentiate between metabolically active and inactive cells. TTC white substrate is enzymatically reduced to red formazan by live cells due to the activity of several dehydrogenases. The change in substrate color is an indirect measure of bacterial metabolic activity.

To mensurate the metabolic activity of biofilm cells, the pins were transferred to 96-well plates with 200 μL/well of fresh BHI medium supplemented with 1% hemin and 0.1% TTC solution. The plates were incubated under anaerobic conditions for 8 h at 37°C. TTC reduction to red formazan was read at 485 nm in a spectrophotometer (Miranda et al., 2019).

#### 2.2.2. Checkerboard DNA–DNA hybridization

The pins coated with 7-day-old biofilms from each group were transferred to Eppendorf tubes containing 100 μL of TE buffer (10 mM Tris–HCl, 1 mM EDTA [pH 7.6]); then, 100 μL of 0.5 M NaOH was added to each tube. The tubes containing the pins and the final solution were boiled for 10 min and the solution was neutralized by adding 0.8 mL of 5 M ammonium acetate. The samples were individually analyzed for the presence and counting of the 33 bacterial species using the DNA–DNA hybridization technique, as previously described (Socransky et al., 1994; Mestnik et al., 2010). Briefly, following sample lysis, the DNA was placed onto a nylon membrane using a Minislot device (Immunetics, Cambridge, United States) and fixed onto the membrane at 120°C for 20 min. Next, the membrane was placed in a Miniblotter 45 (Immunetics). Digoxigenin-labeled whole genomic DNA probes of the 33 bacterial species were hybridized in each lane of the Miniblotter. Following hybridization, the membranes were washed, and DNA probes were detected using a specific antibody to digoxigenin conjugated with alkaline phosphatase. The signals were detected using the AttoPhos substrate (Amersham Life Sciences, Arlington Heights, United States), and the data were obtained in the Typhoon Trio Plus program (Molecular Dynamics, Sunnyvale, United States). Two lanes in each membrane contained the standards with 1 × 10^5^ and 1 × 10^6^ cells of each strain. The signals were converted into absolute counts via comparison with the standards on the same membrane. The measurements of the experimental groups were compared against those of the negative and positive controls. Counts below the method detection limit (1 × 10^4^) were considered zero (Socransky et al., 1994; Miranda et al., 2019).

#### 2.2.3. Statistical analysis

Data from the biofilm’s metabolic activity test were statistically analyzed using Analysis of Variance (ANOVA) followed by Tukey’s test. The results of the Checkerboard DNA–DNA Hybridization were statistically analyzed using Kruskal-Wallis followed by Dunn’s *post hoc* (*p* ≤ 0.05).

## 3. Results

**Figure 2:**
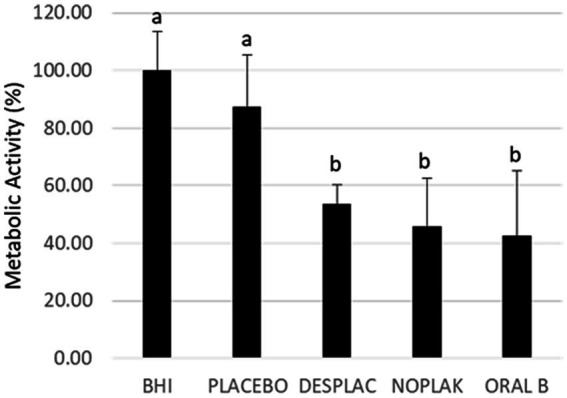
Mean and standard deviation of the mean of the biofilms’ metabolic activities treated with the different agents in experiment A. The metabolic activity of the biofilm treated with the culture medium was considered 100%. Different letters mean a statistically significant difference using ANOVA, followed by Tukey’s test (*p* ≤ 0.05).

**Figure 3:**
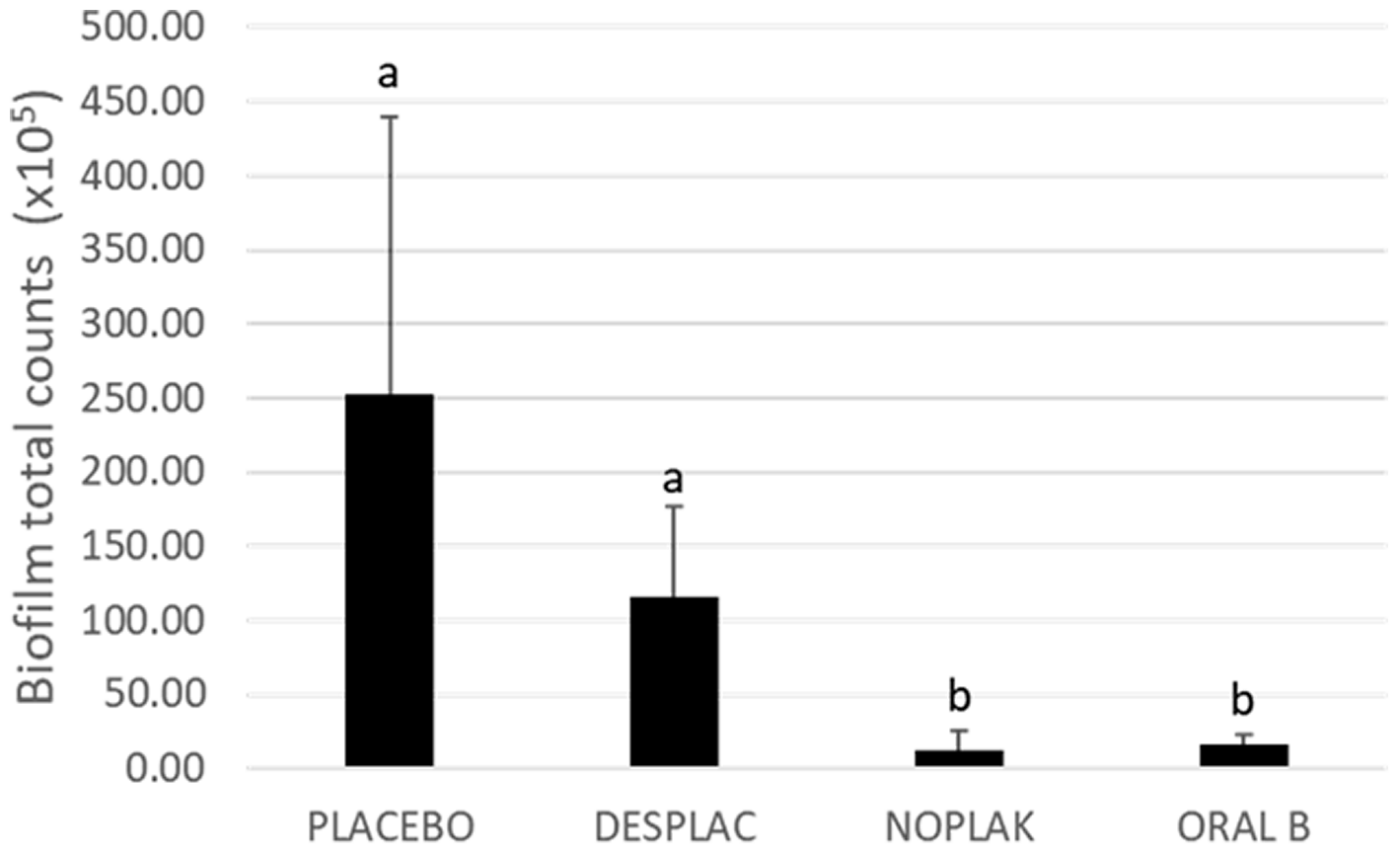
Mean and standard deviation of total counts of all bacterial species in experiment A, analyzed using Checkerboard DNA–DNA Hybridization. Different letters mean a statistically significant difference performed using the Kruskal-Wallis test followed by Dunn’s *post hoc* test (*p* ≤ 0.05).

**Figure 4:**
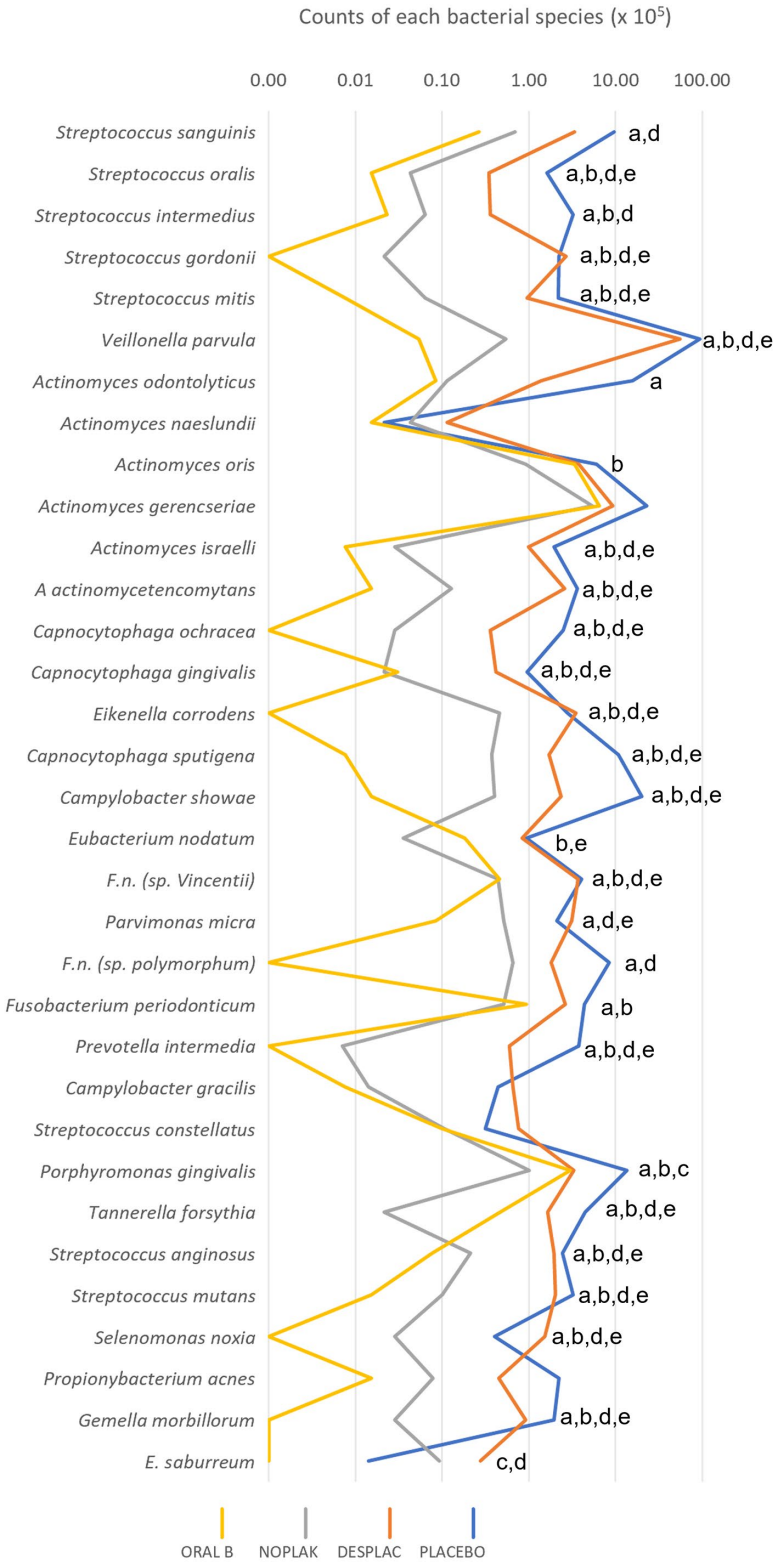
Mean counts of each of the bacterial species present in the biofilms of experiment A. Statistical analysis was performed using the Kruskal-Wallis test followed by Dunn’s *post hoc* test (*p* ≤ 0.05). The letter “a” represents the statistical difference between placebo and Oral B; letter “b” represents the statistical difference between placebo and Noplak; letter “c” represents the statistical difference between placebo and Desplac®; letter “d” represents the statistical difference between Desplac® and Oral B, and letter “e” represents the statistical difference between Desplac® and Noplak.

The analysis of data from Experiment A is shown in Figures 2–4. Figure 2 shows that the metabolic activity of the Desplac® product was statistically similar to Noplak (chlorhexidine + cetylpyridinium chloride) and the Oral B toothpaste (Stannous Fluoride), while the three treatments were statistically better than the placebo group.

Figure 3 shows the total count of all species present in the biofilm subgingival model. Noplak and Oral B reduced total biofilm counts by more than 90% when compared to placebo and Desplac® treated biofilms (*p* ≤ 0.05). In addition, Desplac® and placebo behaved similarly in reducing the total count of bacteria present in biofilms (*p* = 0.07).

Figure 4 shows the individual mean count of each bacterial species included in the biofilm formation evaluated by Checkerboard DNA–DNA Hybridization. The Noplak product reduced the count of 23 bacterial species, the Oral B dentifrice of 25 species, and Desplac® of two species when compared to the placebo-treated biofilms (*p* ≤ 0.05). It is noteworthy that Noplak, Oral B toothpaste, and Desplac® reduced the *P. gingivalis* count demonstrating specific action on key bacteria for the development and progression of periodontal disease.

**Figure 5:**
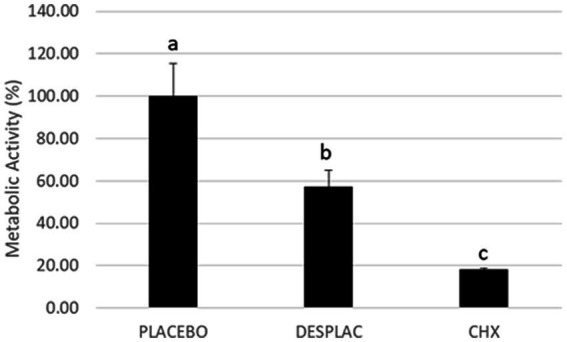
Mean and standard deviation of the mean of the biofilms’ metabolic activities treated with the different agents in experiment B. The metabolic activity of the biofilm treated with the culture medium was considered 100%. Different letters mean a statistically significant difference using ANOVA, followed by Tukey’s test (*p* ≤ 0.05).

Regarding Experiment B, Figure 5 shows that Desplac® statistically reduced biofilm metabolic activity when compared to placebo by about 45% (*p* ≤ 0.05), but Chlorhexidine Gel (0.12%) showed the best inhibition of metabolic activity reducing it by more than 80% (*p* ≤ 0.05).

**Figure 6:**
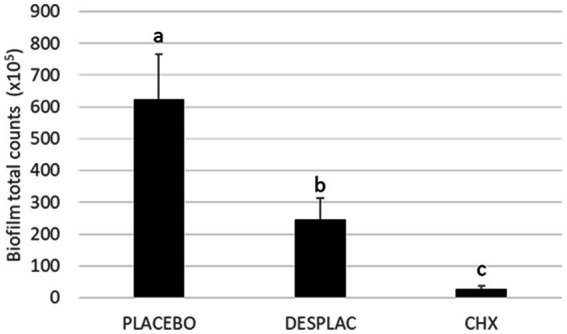
Mean and standard deviation of total counts of all bacterial species in experiment B, analyzed using Checkerboard DNA–DNA Hybridization. Different letters mean a statistically significant difference performed using the Kruskal-Wallis test followed by Dunn’s *post hoc* test (*p* ≤ 0.05).

Figure 6 presents the total count of all bacterial species present in the biofilm model. Desplac® statically reduced the total biofilm count when compared to the placebo by about 59%. However, the chlorhexidine gel (0.12%) showed the best reduction in the total biofilm count, about 89% (*p* ≤ 0.05).

**Figure 7:**
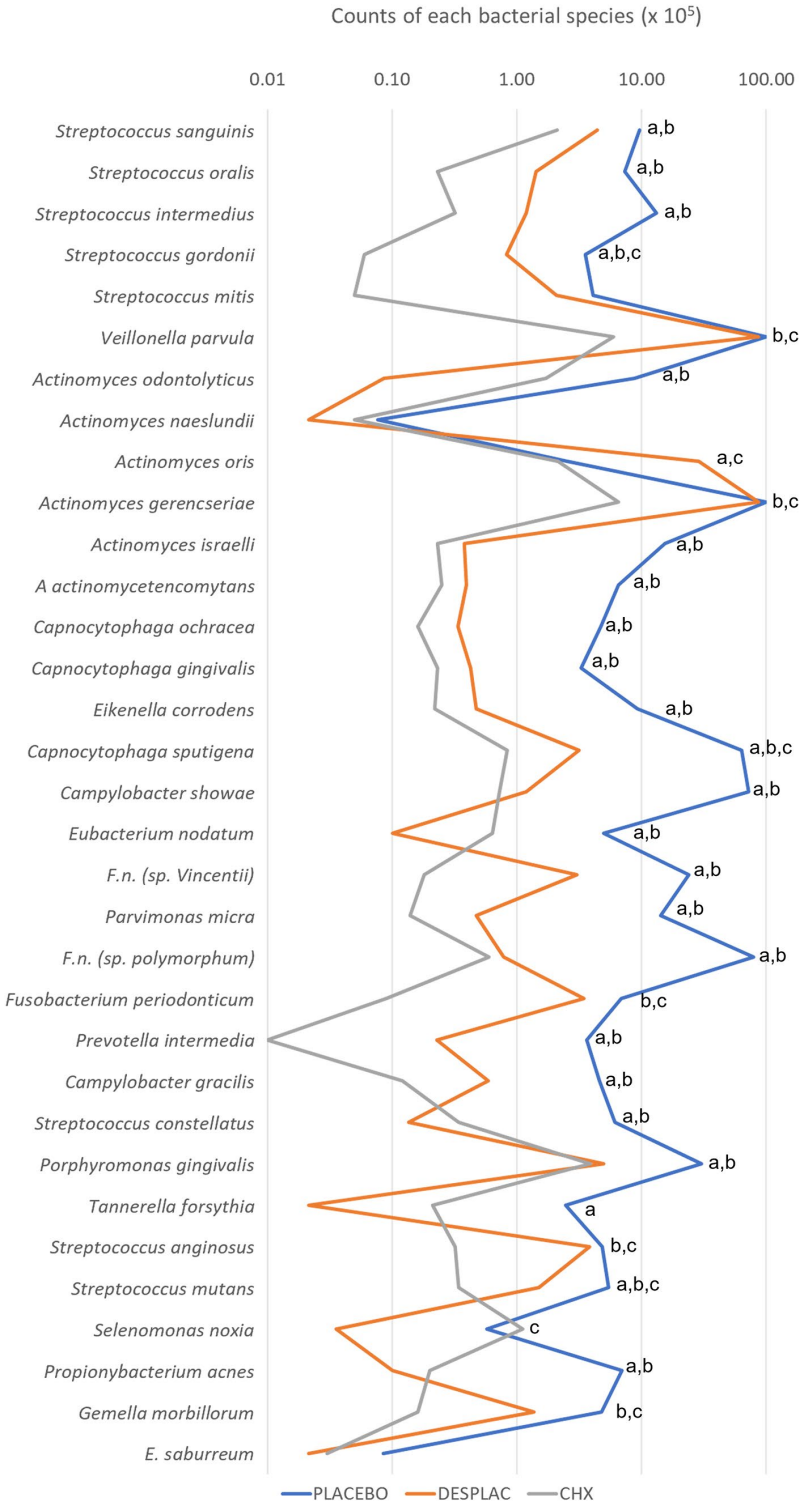
Mean counts of each of the bacterial species present in the biofilm of experiment B. Statistical analysis was performed using the Kruskal-Wallis test followed by Dunn’s *post hoc* test (*p* ≤ 0.05). Letter “a” represents the statistical difference between Placebo and Desplac®; letter “b” represents the statistical difference between Placebo and Chlorhexidine Gel; letter “c” represents the statistical difference between Desplac® and Chlorhexidine Gel.

Figure 7 shows the individual mean count of each bacterial species included in the biofilm formation evaluated by Checkerboard DNA–DNA Hybridization. Chlorhexidine Gel (0.12%) reduced the count of 27 species while Desplac® reduced the count of 24 different bacteria in relation to the placebo group (*p* ≤ 0.05), highlighting *Fusobacterium nucleatum polymorphum*, *Prevotella intermedia,* and *P. gingivalis*, all recognized periodontal pathogens. Only Desplac® was able to reduce *T. forsythia* counts (Figure 7).

## 4. Discussion

The success of periodontal treatment is directly related to an ecological change in the biofilm, making its microbial profile more compatible with periodontal health. This leads to an improvement in periodontal clinical parameters (Cugini et al., 2000; Feres et al., 2015). The therapy known as the gold standard is scaling and root planing. However, not all individuals are able to maintain the benefits achieved with such treatment in the long term (Cugini et al., 2000; Carvalho et al., 2005; Feres et al., 2015). The fact that the standard therapeutic proposal does not reach the periodontopathogens found throughout the mouth—including supragingival biofilm and those in other oral niches, such as the tongue, oral mucosa, and saliva—is one of the reasons for therapeutic failure (Socransky and Haffajee, 2002).

According to the current concept of periodontitis, a dysbiotic microbial community is pointed out as responsible for disease initiation (Hajishengallis and Diaz, 2020). Thus, the red complex members still possess a crucial role in the development of the disease. Among them, *P. gingivalis* and *T. forsythia* are the most studied microorganisms. Both have been proposed as targets to prevent oral microbiome dysbiosis since its incidence may contribute to the shift from healthy to diseased-associated biofilm (Hoare et al., 2021). Therefore, Desplac’s® effects on these bugs are outstanding.

*Porphyromonas gingivalis* has been indicated as the keystone pathogen in periodontal disease since this bacterium produces several virulence factors (for example, gingipains and FimA) with properties to subvert the human immune response, including neutrophils, macrophages, and complement system (Hajishengallis, 2021). Besides its role in periodontitis, *T. forsythia* may be relevant in peri-implantitis pathogenesis. This microorganism was found at more elevated levels in dental implant replacements in contrast with the adjacent tooth, and its presence is correlated with the increase in severity of peri-implantitis (Eckert et al., 2018; Eick et al., 2019).

Healthy sites in individuals with periodontal disease have higher proportions of pathogens when compared to those without the disease (Feres et al., 2015). Hence, the search for anti-infective therapies capable of enhancing the clinical results achieved is constant. In this context, the benefits attained with the use of chlorhexidine stand out among several scientific studies (Carvalho et al., 2005; Mestnik et al., 2010; Feres et al., 2012), and for this reason, it was the chemical agent of choice to represent the positive control group in the experiments of this study.

The problem with the continued use of chlorhexidine mouth rinses is the possibility of developing adverse effects. The most reported in the literature are extrinsic pigmentation of teeth, tongue, mucous membranes and restorations, taste alteration, burning sensation, supragingival calculus formation, and less frequent cases of allergy (Keni et al., 2012; James et al., 2017). For this reason, formulations with other active ingredients have been described in the literature in an attempt to show similar benefits, but with less frequent adverse effects associated with the use of chlorhexidine. Special emphasis can be given to the potential of natural products. Currently, there is a relevant proportion of the world population that searches for cosmetic oral hygiene products (toothpaste and mouthwash) with this profile. The antimicrobial activity of the natural agents propolis, Aloe vera, green tea, cranberry, and calendula is already evidenced in the scientific literature. However, to date, this activity evaluated in a combined way as in the commercial product Desplac® is unprecedented in the literature.

In this direction, the green propolis produced in the South region of Brazil was the first to be recognized for its antimicrobial potential. Recently, it was found to impair gut microbiota dysbiosis by enhancing the Bacteroidetes/Firmicutes proportion in an animal study (Okamura et al., 2022). In addition, the baccharin, one of its biocompounds, has shown a possible antimicrobial mechanism of action on *P. gingivalis*. As an antimicrobial mechanism, this compound induces membrane depolarization so to increase membrane permeability leading to bacterial death (Yoshimasu et al., 2018). The apigenin, found in green propolis, has been shown to inhibit the development of *Candida albicans* (Cheah et al., 2014) and *Streptococcus mutans* (Jeon et al., 2011).

Another natural agent is Aloe vera or *Aloe barbadensis*. It is also an option in toothpaste considering its antimicrobial potential on oral microorganisms, such as *S. mutans* and *C. albicans*, and improvement in plaque index comparable to those obtained with products with triclosan in their composition (Lee et al., 2004; Pradeep et al., 2012; Vajrabhaya et al., 2022). The Aloe vera main components are aloin A, aloin B, aloesin, aloe-emodin, aloeresin D, orientin, cinnamic acid, and chlorogenic acid (Solaberrieta et al., 2022). Among them, the antibacterial mechanism of aloe-emodin was determined on *Staphylococcus epidermidis*. The compound provokes abnormalities in *S. epidermidis* morphology and ruins membrane permeability (Li et al., 2021).

Several components of green tea can also promote health benefits. Mazur et al. (2021) demonstrated through a systematic review that clinical periodontal parameters were found to be positively affected by green tea. Chemical analysis of green tea revealed the presence of some phenolic compounds (rutin, quercetin, and chlorophyll) and four main catechins: epicatechin (EC), epicatechin-3-gallate (ECG), epigallocatechin (EGC), and epigallocatechin-3-gallate (EGCG); the latter being the most active and abundant among them (Ku et al., 2010; Reygaert, 2018; Kolackova et al., 2020). More recently, Kong et al. (2022) published a literature review showing the antimicrobial activity of epigallocatechin-3-gallate, one of the green tea’s compounds as mentioned, in the microbiota associated with oral diseases. The antimicrobial effect was evident for *P. gingivalis*, *A. actinomycetemcomitans*, *P. intermedia,* and *F. nucleatum*. EGCG damages the *P. gingivalis* membrane and cellular wall preventing biofilm formation and ruining the pre-formed biofilm. Regarding *A. actinomycetemcomitans*, EGCG inhibits a relevant virulence factor, the leukotoxin that is associated with the impairment of human macrophages.

Cranberry has bioactive agents such as proanthocyanidins (propelargonidin, procyanidin, and prodelphinidin) that characterize this natural product as beneficial for health (Zhao et al., 2020). In dentistry, Polak et al. (2013) showed the potential protective and/or preventive effect of cranberry on *P. gingivalis* and *F. nucleatum*-induced periodontitis in mice. Galarraga-Vinueza et al. (2020) demonstrated that proanthocyanidins, known to inhibit oral biofilm adherence and for their anti-inflammatory effect, could potentially neutralize the destructive inflammatory response of macrophages. These compounds do not interfere with *P. gingivalis* growth; however, they inhibit many virulence factors related to *P. gingivalis* adhesion, such as collagenases, proteinases, and other proteins associated with *P. gingivalis*’s attachment to periodontal tissue, with subsequently smaller bacterial biofilm formation (Nawrot-Hadzik et al., 2021a). In recent years, the medicinal potential of *Calendula officinalis* has encouraged scientific studies in dentistry especially on topics involved in the treatment of periodontitis and peri-implantitis (Lima et al., 2017; Alexandre et al., 2018; Tanideh et al., 2020). Although calendula presents distinct classes of well-known antimicrobial compounds in its composition, such as triterpenoids, flavonoids, quinones, tannins, coumarins, and phenolic acids, the literature on calendula’s antimicrobial activity is scarce., The only report found demonstrated that a calendula-based dentifrice did not present an antimicrobial effect on *A. viscosus, C. albicans, L. casei, S. mitis, S. mutans,* *S. oralis, S. sanguis, S. sobrinus,* and clinically isolated *C. albicans, S. mitis, S. mutans, S. oralis, S. sanguis, S. sobrinus,* and *Lactobacillus* spp. (Modesto et al., 2000).

It is interesting to observe how the agent’s contact time with the biofilm improved the antibacterial effect. The unique 12-h treatment of an established biofilm reduced a more significant number of species than two daily treatments of 1 min during the biofilm formation. Usually, an established biofilm is a more complex challenge for antimicrobial agents than a biofilm in formation. However, probably due to the time of contact, Desplac® reduced a larger number of species in experiment B (12-h treatment of an established biofilm).

Considering the design of this study, it is important to point out that experiments A and B correspond to the uses recommended by the manufacturer in accordance with ANVISA’s authorization for the commercialization of Desplac®. It is important to note that chlorhexidine gel does not have an indication to be used overnight as Desplac®. However, due to the absence of a positive control with this kind of indication, chlorhexidine gel was kept as a positive control of experiment B due to its excellent antimicrobial properties. In addition, limitations of the biofilm model include the semi-quantitative characteristic of the checkerboard and the absence of *Treponema denticola* since this bug is also a member of the red complex (Socransky et al., 1998). Moreover, a possible improvement of the present biofilm model may include further examination, such as confocal microscopy, that would allow the assessment of the biofilm portion structure, bacteria biomass, and exopolysaccharide amount. Currently, confocal microscopy analysis is prevalent for caries-related monospecies biofilms but not periodontal ones. Therefore, future studies should consider improving the existing knowledge by evaluating dyes for confocal analysis of periodontitis-related multispecies biofilms (Torrez et al., 2023). The analysis of the data obtained in this laboratory research showed promising results related to antimicrobial activity in a multispecies subgingival biofilm. Thus, it was possible to conclude that the combination of natural agents present in the commercial product Desplac® was able to inhibit the biofilm development and disrupt the mature subgingival biofilm, highlighting its effect on *T. forsythia* counts. Although the present subgingival multispecies biofilm was revealed as a good model for the initial analysis of novel antibacterial agents, it is still necessary to carry out randomized controlled clinical studies in order to confirm whether the microbiological benefits observed here will be able to support the periodontal clinical condition associated with health.

## Data availability statement

The raw data supporting the conclusions of this article will be made available by the authors, without undue reservation.

## Author contributions

LF, BB-S, EO, and AB: conceptualization. LF, BB-S, MM, and MF: methodology. BB-S, MM, and KK: data analysis. BB-S, LF, and FG: resources. LF, BB-S, KK, and FG: writing—original draft preparation. LF, BB-S, and MF: writing—review and editing. All authors contributed to the article and approved the submitted version.

## Funding

This study was funded by the Coordination for the Improvement of Higher Education Personnel (CAPES, Brazil) through the PROEX program (grant number 0475/2019, process number 23038.005614/2019-74), CNPq - National Council for Scientific and Technological Development, Brazil (L.C.F., grant #313647/2021-6) and by the Sysplac Company for the acquisition of the necessary material to carry out the laboratory experiments.

## Conflict of interest

EO was the owner of Sysplac Company that produces Desplac product.

The remaining authors declare that the research was conducted in the absence of any commercial or financial relationships that could be construed as a potential conflict of interest.

## Publisher’s note

All claims expressed in this article are solely those of the authors and do not necessarily represent those of their affiliated organizations, or those of the publisher, the editors and the reviewers. Any product that may be evaluated in this article, or claim that may be made by its manufacturer, is not guaranteed or endorsed by the publisher.
